# The relationship between hypertriglyceridemic wait-to-height ratio and hypertension–diabetes comorbidity among older adult

**DOI:** 10.3389/fpubh.2023.1292738

**Published:** 2023-12-07

**Authors:** Ping Zhang, Yangyang Xiong, Menghan Chen, Huaide Zhang, Nan Sun, Fan Wu, Jiayu Yang, Yongcheng Ren

**Affiliations:** ^1^Department of Rehabilitation Medicine, Zhumadian Central Hospital, Zhumadian, China; ^2^Institute of Health Data Management, Huanghuai University, Zhumadian, China

**Keywords:** hypertriglyceridemic waist-to-height ratio, hypertension-diabetes comorbidity, older adult, public health, epidemiology

## Abstract

**Objective:**

Limited information is available on the effect of hypertriglyceridemic waist-to-height ratio (HTHWH) and hypertension–diabetes comorbidity (HAD) in older adult people. We aimed to explore the relationship between HTHWH and HAD for the co-management of hypertension and diabetes mellitus in the older adult.

**Methods:**

A cross-sectional study, randomized cluster sampling from 10 community health service centers, and multivariate logistic regression were used in this study. A total of 3,501 participants aged 65 years or older recruited between January 2019 and December 2019 completed the study.

**Results:**

Among 3,501 participants, the median age was 69.96 years, and 42.50% were men. A total of 1,207 subjects were in the HTHWH group, and the prevalence rate of HAD was 17.23% in this group. Multivariate logistic regression analysis showed that, as compared with the normal group, the risk of HAD in the HTHWH group increased by 2.05 times (OR = 3.05, 95% CI: 2.06–4.51). The risks of hypertension or diabetes mellitus (HOD), hypertension, and diabetes mellitus were also increased in the HTHWH group, with their ORs (95%CIs) being 1.82 (1.44–2.29), 1.73 (1.38–2.17), and 2.28 (1.66–3.13), respectively.

**Conclusion:**

HTHWH significantly increases the risk of HAD and can be used as a reliable tool to screen the high-risk population for HAD.

## 1 Introduction

Hypertension and diabetes are important risk factors for cardiovascular disease and premature death (1, 2). With the change in lifestyle and the acceleration of the aging process, hypertension–diabetes comorbidity (HAD) is prevalent among the older adult (3). Based on the monitoring data of chronic diseases and risk factors in China in 2018, the prevalence of HAD reached 13.5, 17.7, and 20.6% among people aged 55–64, 65–74, and ≥75, respectively (4). It is critical to explore the risk factors of HAD and take measures to reduce its risk and disease burden. As a special metabolic type, hypertriglyceridemic waist (HTGW) is characterized by abdominal fat accumulation and triglyceride (TG) elevation. Previous studies have shown that HTGW can increase the risk of hypertension, diabetes, and cardiovascular disease (5–8), but there is little evidence about the relationship between HTGW and HAD. In addition, considering that waist-to-height ratio (WHtR) can better evaluate abdominal obesity than waist circumference (WC) (9), hypertriglyceridemic WHtR (HTHWH) may better reflect the risk of HAD. Therefore, based on the physical examination data of the older adult in 10 community health service centers, this study analyzed the relationship between HTHWH phenotype and HAD in the older adult population over 65 years of age, as well as provided evidence based on the natural population for the prevention and management strategies for older adult groups with HAD.

## 2 Materials and methods

### 2.1 Sampling and study population

**Figure 1:**
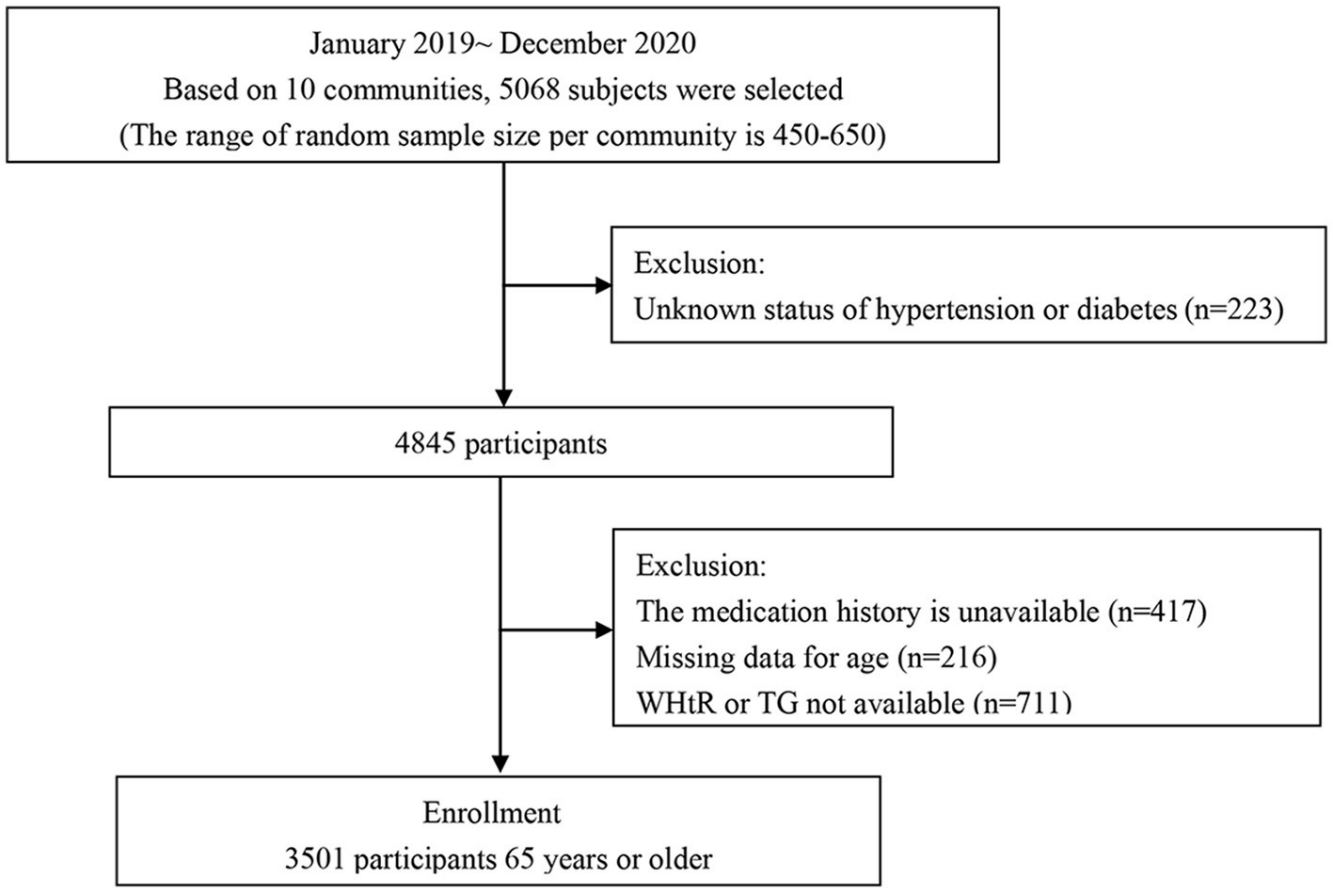
Composition of the HTHWH phenotype and prevalence of HAD.

A total of 5,068 permanent residents aged 65 years or older were selected from 10 communities for this study using a random cluster sampling method from January 2019 to December 2020, with the local community health service center as the basic unit, and the range of random sample size per community is 450–650. After excluding those with unknown hypertension or diabetes status and those with missing information on age, WHtR, TG, and medication history, a total of 3,501 subjects were included in this study (Figure 1).

### 2.2 Main research contents

The questionnaire information includes demographic characteristics: age, gender, education level, and marital status; behavioral risk factors: smoking, drinking, physical activity, and dietary habits; family history of disease: family history of hypertension, diabetes, and other cardiovascular and cerebrovascular diseases; anthropometric parameters: height, weight, WC, blood pressure, and medication history; and biochemical indicators: fasting blood glucose, triglyceride (TG), and total cholesterol (TC).

### 2.3 Definitions of key variables

According to whether TG ≥ 1.7 mmol/L and WHtR ≥ 0.5, the subjects were divided into four groups: normal TG and normal WHtR group (NTNWH), normal TG and high WHtR group (NTHWH), high TG and normal WHtR group (HTNWH), and high TG and high WHtR group (HTHWH). Hypertension is defined as systolic blood pressure ≥140 mmHg and/or diastolic compression pressure ≥90 mmHg and/or taking antihypertensive drugs. Diabetes is defined as fasting blood glucose ≥7.0 mmol/L and/or taking hypoglycemic drugs. HAD is defined as the subject suffering from hypertension and diabetes at the same time.

### 2.4 Statistical analysis

Continuous variables were described by median (interquartile range), and the Kruskal–Wallis test was used to compare different HTHWH phenotypes. Categorical variables were described by frequency (percentage), and the chi-square test was used to compare between groups. Multivariate logistic regression was used to analyze the relationship between the HTHWH phenotype and HAD. Three models were constructed: Model 1, adjusting age and sex; Model 2: further adjusting education level, marital status, smoking status, drinking status, diet, and physical activity level; Model 3: adjusting family history of hypertension with diabetes, TC, and prevalence of cardiovascular disease or malignant tumor. Based on the above model, we also evaluated the relationship between the HTHWH phenotype and hypertension or diabetes (HOD), hypertension, and diabetes. All analyses were performed by SPSS 23.0 statistical software, with a two-sided test level of α = 0.05.

## 3 Results

**Figure 2:**
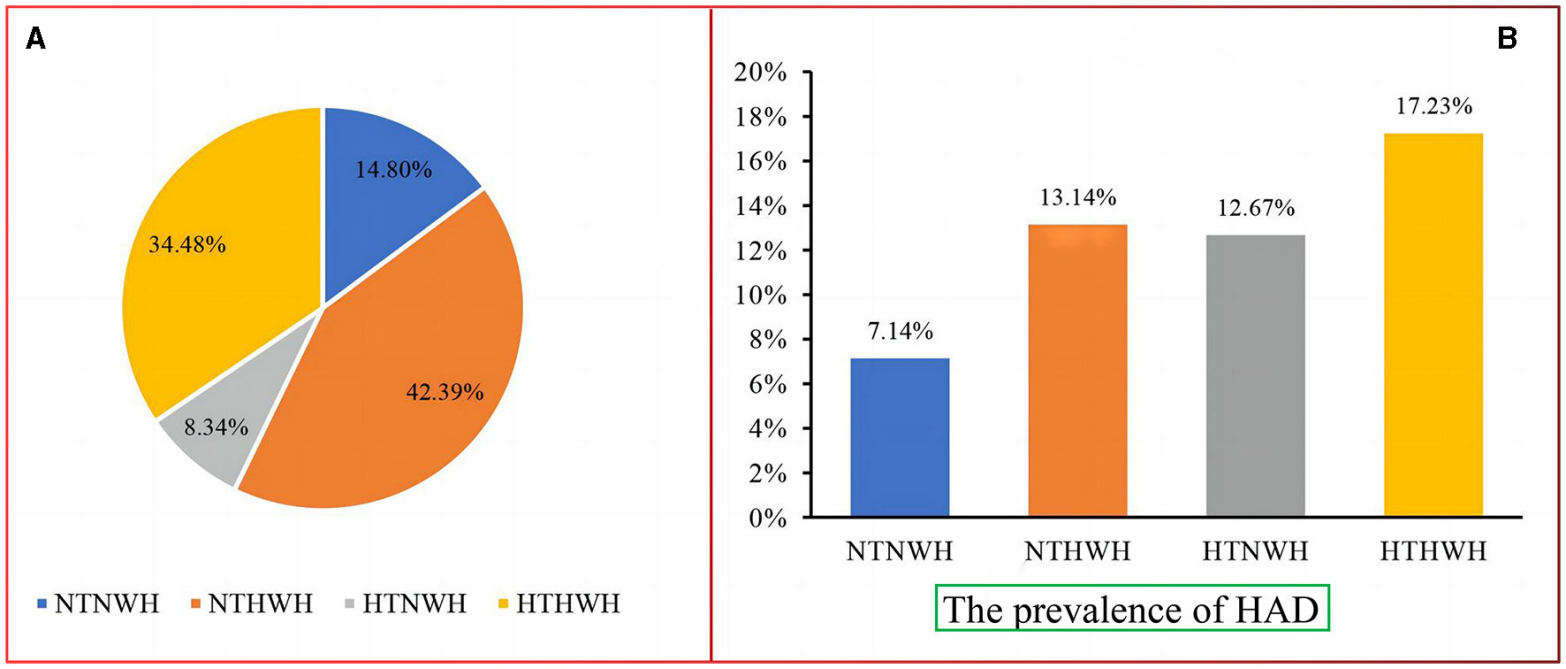
**(A)** Composition of the HTHWH phenotype and **(B)** prevalence of HAD. NTNWH, normal triglyceride and normal waist-to-height ratio; NTHWH, normal triglyceride and high waist-to-height ratio; HTNWH, high triglyceride and normal waist-to-height ratio; HTHWH, high triglyceride and high waist-to-height ratio; HAD, hypertension-diabetes comorbidity.

A total of 3,501 subjects (42.50% men) were included in this study, with a median age of 69.96 years. A comparison of characteristics between the four groups is presented in Table 1. Age, sex, education level, smoking status, WC, TG, and TC were different among different phenotypes (*P* < 0.05). NTHWH accounted for the highest proportion, accounting for 42.39%, followed by HTHWH (34.48%), NTNWH (14.80%), and HTNWH (8.34%) (Figure 2A).

**Table 1:** Basic characteristics of subjects with different HTHWH phenotypes.

**Characteristics**	**NTNWH**	**NTHWH**	**HTNWH**	**HTHWH**	***P-value***
Age (IQR, years)	69.71 (7.81)	70.58 (8.04)	68.74 (5.7)	69.57 (6.57)	<0.0001
Male (%)	319 (61.58)	652 (43.94)	142 (48.63)	375 (31.07)	<0.0001
Married (%)	490 (94.59)	1,378 (92.86)	273 (93.49)	1,134 (93.95)	0.2122
High school and above (%)	132 (25.48)	286 (19.27)	80 (27.4)	237 (19.64)	0.0006
Smoking (%)	56 (10.85)	144 (9.84)	17 (5.84)	73 (6.14)	0.0004
Drinking alcohol (%)	5 (0.97)	14 (0.94)	1 (0.34)	15 (1.24)	0.6373
Ideal diet (%)	487 (97.21)	1,373 (94.69)	268 (96.06)	1,140 (95.8)	0.1067
Ideal physical activity (%)	39 (7.53)	158 (10.65)	27 (9.25)	122 (10.11)	0.2217
Family history of diabetes or hypertension (%)	6 (1.16)	25 (1.68)	6 (1.03)	19 (1.57)	0.7476
Waist circumference (IQR, cm)	78.00 (8.00)	88.00 (1.00)	78.00 (6.00)	88.00 (10.00)	<0.0001
Triglyceride (IQR, mmol/L)	1.07 (0.48)	1.16 (0.5)	2.29 (1.07)	2.4 (1.15)	<0.0001
Total cholesterol (IQR, mmol/L)	4.87 (1.33)	4.81 (1.53)	5.42 (1.48)	5.45 (1.61)	<0.0001
History of CVD or cancer (%)	40 (7.72)	123 (8.29)	12 (4.11)	89 (7.37)	0.1026

IQR, interquartile range; NTNWH, normal triglyceride and normal waist-to-height ratio; NTHWH, normal triglyceride and high waist-to-height ratio; HTNWH, high triglyceride and normal waist-to-height ratio; HTHWH, high triglyceride and high waist-to-height ratio; CVD, cardiovascular disease.

The prevalence of HAD in the NTNWH, NTHWH, HTNWH, and HTHWH groups was 7.14, 13.14, 12.67, and 17.23%, respectively (Figure 2B). Compared to NTNWH, the OR (95% CI) of the NTHWH, HTNWH, and HTHWH groups was 1.97 (1.36–2.85), 1.90 (1.18–3.08), and 2.73 (1.89–3.96), respectively, after adjusting for age and sex (Table 2). In Model 3, the risk of HAD in NTHWH, HTNWH, and HTHWH groups increased by 120% (OR = 2. 20, 95% CI: 1.50–3.25), 127% (2.27, 1.38–3.74), and 205% (3.05, 2.06–4.51), respectively.

**Table 2:** Relationship between different HTHWH phenotypes and HAD.

	**Total**	**Cases**	**Model 1**	**Model 2**	**Model 3**
NTNWH	518	37	1.00	1.00	1.00
NTHWH	1,484	195	1.97 (1.36–2.85)	2.21 (1.50–3.25)	**2.20 (1.50–3.25)**
HTNWH	292	37	1.90 (1.18–3.08)	2.21 (1.34–3.63)	**2.27 (1.38–3.74)**
HTHWH	1207	208	2.73 (1.89–3.96)	3.04 (2.05–4.49)	**3.05 (2.06–4.51)**

NTNWH, normal triglyceride and normal waist-to-height ratio; NTHWH, normal triglyceride and high waist-to-height ratio; HTNWH, high triglyceride and normal waist-to-height ratio; HTHWH, high triglyceride and high waist-to-height ratio; HAD, hypertension-diabetes comorbidity. Model 1: Adjust age and sex; Model 2: Adjust Model 1 + education level, marital status, smoking status, drinking status, diet, and physical activity level; Model 3: Adjusted Model 2 +family history of hypertension with diabetes, total cholesterol level, medication history, and prevalence of cardiovascular disease or malignant tumor.

The prevalence of HOD in the NTNWH, NTHWH, HTNWH, and HTHWH groups was 64.29, 71.50, 70.55, and 74.90%, respectively. The prevalence of hypertension was 59.65, 66.51, 63.36, and 69.84%, respectively. The prevalence of diabetes was 11.78, 18.13, 19.86, and 22.29%, respectively. After adjusting for potential confounding factors (Table 3), taking NTNWH as a reference, the risks of HOD and diabetes in the NTHWH, HTNWH, and HTHWH groups increased by 49, 40, 82%, and 80, 99, 128%, respectively; the risk of hypertension in the NTHWH and HTHWH groups increased by 42 and 73%, respectively.

**Table 3:** Relationship between different HTHWH phenotypes and hypertension or diabetes mellitus.

	**Total**	**Cases**	**Model 1**	**Model 2**	**Model 3**
**HOD**					
NTNWH	518	333	1.00	1.00	1.00
NTHWH	1,484	1,061	1.39 (1.13–1.73)	1.50 (1.20–1.87)	**1.49 (1.20–1.86)**
HTNWH	292	206	1.39 (1.02–1.89)	1.37 (1.00–1.89)	**1.40 (1.02–1.92)**
HTHWH	1,207	904	1.72 (1.37–2.17)	1.81 (1.44–2.29)	**1.82 (1.44–2.29)**
**Hypertension**					
NTNWH	518	309	1.00	1.00	1.00
NTHWH	1,484	987	1.34 (1.09–1.65)	1.43 (1.15–1.77)	**1.42 (1.15–1.77)**
HTNWH	292	185	1.23 (0.91–1.65)	1.25 (0.92–1.69)	1.27 (0.94–1.73)
HTHWH	1,207	843	1.64 (1.31–2.04)	1.73 (1.38–2.17)	**1.73 (1.38–2.17)**
**Diabetes mellitus**					
NTNWH	518	61	1.00	1.00	1.00
NTHWH	1,484	269	1.67 (1.24–2.26)	1.81 (1.33–2.47)	**1.80 (1.32–2.46)**
HTNWH	292	58	1.84 (1.24–2.73)	1.97 (1.31–2.96)	**1.99 (1.33–3.00)**
HTHWH	1,207	269	2.15 (1.58–2.91)	2.27 (1.66–3.12)	**2.28 (1.66–3.13)**

HOD, hypertension or diabetes mellitus; NTNWH, normal triglyceride and normal waist-to-height ratio; NTHWH, normal triglyceride and high waist-to-height ratio; HTNWH, high triglyceride and normal waist-to-height ratio; HTHWH, high triglyceride and high waist-to-height ratio. Model 1: Adjust age and sex; Model 2: Adjust Model 1 + education level, marital status, smoking status, drinking status, diet, and physical activity level; Model 3: Adjusted Model 2 + family history of hypertension or diabetes, total cholesterol levels, medication history, and prevalence of cardiovascular disease or malignant tumors.

## 4 Discussion

The HTHWH phenotype is a simple and effective tool for evaluating abdominal obesity and metabolic disorders. This study shows that HTHWH in the older adult is independent of other risk factors and is related to the increased risk of HAD. Correspondingly, previous studies based on the rural population in Henan showed that HTHWH can increase the risk of hypertension and diabetes, respectively, in people younger than 60 years old, while HTHWH has nothing to do with the increased risk of hypertension and diabetes in people older than 60 years old, which may be related to the small number of cases in the older adult (10, 11). Compared with the NTNWH group, the risk of HAD in the HTHWH group increased by 2.05 times, which was higher than that of hypertension alone (0.73 times) or diabetes alone (1.28 times), suggesting that HTHWH may also make individuals suffering from a single disease more susceptible to another disease, which also highlights the necessity of studying comorbidity. Most studies have combined TG and WC to construct HTGW and explore its relationship with hypertension and diabetes (9, 12–14). We constructed HTGW and found that HTGW can increase the risk of HAD, HOD, hypertension, and diabetes by 1.69 times, 0.59 times, 0.55 times, and 1.05 times, respectively (Supplementary Table S1), which is lower than the corresponding 2.05 times, 0.82 times, 0.73 times, and 1.28 times of HTHWH, which indicates that compared with HTGW, HTHWH can better predict the risk of hypertension and diabetes comorbidity.

In this study, more than one-third of the people are in HTHWH state, which is significantly higher than the HTGW prevalence of 27.85% in the rural older adult in Henan, 27.1% in the older adult community in western Brazil, 15.93% in China's health and pension follow-up survey cohort study, and 18% of adults worldwide (8, 13, 15). The prevalence of HTHWH in both domestic and foreign populations is quite high, especially in the older adult. Considering the high prevalence rate of HTHWH and the great disease risk caused by it, it is of great significance to evaluate the phenotype of HTHWH in the older adult to screen out people with high comorbidity risk so they can reduce the risk and burden of comorbidity between hypertension and diabetes.

The strength of this study is that the combined TG and WHtR constructed the HTHWH phenotype to assess abdominal obesity and metabolic disorder status and explored the effect of HTHWH on HAD in the older adult population. The limitation is that because this is a cross-sectional study of the older adult population, extending the conclusion to the entire population is limited, and it has to be validated in a cohort study with a larger sample.

In summary, this study found that the prevalence of HTHWH in the older adult population is high, and it leads to a higher comorbidity risk of hypertension and diabetes. This study suggests that in the older adult, we can screen the high-risk groups of HAD by evaluating HTHWH status and then better manage the comorbidity of chronic diseases.

## Data availability statement

The raw data supporting the conclusions of this article will be made available by the authors, without undue reservation.

## Ethics statement

This study was conducted according to the guidelines laid down in the Declaration of Helsinki and all procedures involving human subjects were approved by the Ethics Committee of Huanghuai University. The studies were conducted in accordance with the local legislation and institutional requirements. The participants provided their written informed consent to participate in this study.

## Author contributions

YR: Formal analysis, Funding acquisition, Methodology, Writing—original draft, Writing—review & editing. YX: Formal analysis, Investigation, Methodology, Writing—original draft. PZ: Data curation, Investigation, Writing—review & editing. MC: Data curation, Investigation, Writing—review & editing. HZ: Data curation, Investigation, Writing—review & editing. NS: Data curation, Investigation, Writing—review & editing. FW: Data curation, Investigation, Writing—review & editing. JY: Data curation, Investigation, Writing—review & editing.
