# Supplementary material for: The relationship between hypertriglyceridemic wait-to-height ratio and hypertension–diabetes comorbidity among older adult
Source: Front Public Health. 2023 Dec 7;11:1292738. doi: 10.3389/fpubh.2023.1292738 (PMC10733473; doi:10.3389/fpubh.2023.1292738)
Supplement: Supplementary file 1 [file Table_1.DOCX]

**Table S1. Relationship between different HTGW phenotypes and hypertension and diabetes**

|  | HAD | HOD | Hypertension | Diabetes |
| --- | --- | --- | --- | --- |
| NTNW | 1.00 | 1.00 | 1.00 | 1.00 |
| NTGW | 2.14（1.56-2.94） | 1.29（1.05-1.59） | 1.26（1.03-1.54） | 1.76（1.35-2.29） |
| HTNW | 2.23（1.56-3.18） | 1.34（1.05-1.71） | 1.25（0.99-1.58） | 1.92（1.43-2.59） |
| HTGW | 2.69（1.94-3.73） | 1.59（1.27-1.99） | 1.55（1.25-1.93） | 2.05（1.55-2.70） |

Note: NTNW, normal triglyceride and normal waist circumference; NTGW, normal triglyceride and high waist circumference; HTNW, high triglyceride and normal waist circumference; HTGW, high triglyceride and high waist circumference; HAD: hypertension-diabetes comorbidity; HOD: hypertension or diabetes.

Adjust age, sex, education level, marital status, smoking status, drinking status, diet and physical activity level, family history of hypertension with diabetes, total cholesterol level, and prevalence of cardiovascular disease or malignant tumor.

调整年龄、性别、受教育水平、婚姻状况、吸烟状况、饮酒状况、膳食情况、体育活动水平、高血压合并糖尿病家族史、总胆固醇水平和心血管疾病或恶性肿瘤患病情况
